# The phenomenology of attentional control: a first-person approach to contemplative science and the issue of free will

**DOI:** 10.3389/fpsyg.2024.1349826

**Published:** 2024-03-12

**Authors:** Terje Sparby, Dirk Cysarz, David Hornemann v. Laer, Friedrich Edelhäuser, Diethard Tauschel, Ulrich W. Weger

**Affiliations:** ^1^Department of Psychology and Psychotherapy, Witten/Herdecke University, Witten, Germany; ^2^Integrated Curriculum for Anthroposophic Psychology (ICURAP), Witten/Herdecke University, Witten, Germany; ^3^Rudolf Steiner University College, Oslo, Norway; ^4^Institute of Integrative Medicine, Witten/Herdecke University, Witten, Germany; ^5^Integrated Curriculum for Anthroposophic Medicine (ICURAM), Witten/Herdecke University, Witten, Germany; ^6^WittenLab. Zukunftslabor Studium Fundamentale, Witten/Herdecke University, Witten, Germany

**Keywords:** attention, freedom, first-person perspective, introspection, contemplative science

## Abstract

There are two basic aspects of attentional control. The ability to *direct attention* toward different objects is typically experienced as a fundamental indicator of attentional freedom. One can control what one attends to and directing attention is a relatively simple task. In contrast, *sustaining attention* on a chosen object proves to be difficult as mind-wandering seems to be inevitable. Does the problem of sustaining attention, mean that we are fundamentally unfree? We discuss this issue in light of an introspective study of directing and sustaining attention, looking specifically into the question of whether it is possible to experience the source of attention, i.e., the subject enacting freedom through attention. The study involved six persons performing different attention tasks over the course of about a month. Common experiences and contrasting reports are presented. This forms the basis for a discussion of the method of introspection and in particular of how to approach conflicting reports.

## 1 The theory, practice and challenges of introspective research in relation to freedom and attention

We will begin by considering the idea of introspective research and how it can be used to investigate freedom and attention. Section 1.1 below gives an overview over the idea of introspective research and some problems it faces. A strong and weak conception of introspection is introduced, representing a high and low degree of certainty, respectively. Both conceptions are problematic and it is suggested that the key challenge for introspective research is to develop methods that yield a moderate degree of certainty. Section 1.2 presents the problem of free will and asks: How can introspective research be made use of to inform the discussion of free will? Directing and sustain attention is brought in relation to the issue of freedom and the question of the source of attention, i.e., who or what is the source of attentional control.

### 1.1 The idea of introspective research

First person methods, such as introspection (Weger and Wagemann, 2015a), phenomenology (Giorgi, 2009), and descriptive psychology (Brentano, 1982), are typically approached with a certain skepticism with regards to their status as viable forms of scientific inquiry. More or less a century has passed since the early attempts at creating a foundation of introspection (Beenfeldt, 2013) and phenomenology (Farber, 2006) as scientific disciplines. Despite numerous attempts (Giorgi, 2009), no specific first person methodology has been commonly recognized to have the necessary rigor to become part of the standard repertoire of scientific methods. However, what often goes unnoticed, and what proponents of first person methods tend to point out, is that many scientific disciplines rely on accurate first person accounts. For instance, the correlation between physiological processes and subjective experiences not only depends on accurate physical measurements, but also on the person having those experiences being able to report accurately on them. All forms of inquiry involving first person experiences will therefore depend on the rigor of the methods used to investigate such experiences. Current studies that make use of naïve first person reports may, for instance, overlook ambiguities inherent in lived experience or inaccurate reports may be used to support specific hypotheses that could have been challenged by the reports of subjects who have undergone introspective training. The case of the rubber hand illusion is an example of the former (Valenzuela Moguillansky et al., 2013) the Libet-experiment is a good example of the latter (Jo et al., 2015). Furthermore, introspective training such as meditation is known to increase the accuracy of reports (Fox et al., 2012).

In other words, the general lack of rigorous first-person methods can be seen as a fundamental challenge to the whole field of psychology and neighboring disciplines such as contemplative science that either implicitly or explicitly rely heavily on first person accounts. In the context of this article, we understand contemplative science as a combination of first-person and third-person approaches when systematically investigating what the mind experiences through meditative practice (Sparby, 2017). Since the mind first and foremost is a first-person phenomenon, first-person approaches are the ones that are most suited to investigate meditative experiences. However, since first-person approaches are less developed than third person ones, contemplative science depends specifically on the development of first-person approaches in order to advance as a field. Unless terms referring to first-person meditation experiences are rigorously defined, the validity of empirical research on such experience is strongly limited (Sparby and Sacchet, 2022). For example, research on advanced states of meditation, such as advanced concentrative absorption meditation, sometimes referred to as jhāna (Yang et al., 2023), will have limited validity of so-long as “jhāna” refers to different sets of first-person experiences, which is arguably the case (Sparby and Sacchet, 2024).

What also often goes unnoticed is that scientific theorizing itself depends on a form of introspection. When, for instance, someone is devising a theory or falsifying a hypothesis, they make use of certain inferential structures that are not derived from the external senses. The structures of inferences, such as modus ponens, or pure concepts or mathematical insights, enter conscious awareness through an act of introspection. Typically, there is no awareness of this act since the focus is on the content rather than on the act. Nonetheless, theorizing involves acts of introspection. Interestingly, one does not ask *how many* agree that a mathematical equation or valid inference is true; the truth of such matters is gleaned directly from the content. In contrast, empirical psychological studies depend on having a high enough sample size in order to provide convincing results. Phenomenology has, since its beginnings, been concerned with the possibility of finding the essential structure of something, through for instance what Husserl called *Wesensschau* or eidetic intuition (Husserl et al., 2001), *without* investigating the degree of intersubjective agreement on the results of such an intuition. And this is a central issue when evaluating the truthfulness of introspection as well. Is it possible to perform some mental procedure that will give insight into certain characteristics of psychological phenomena in such a way that the question of how many agree that the phenomena exhibit these characteristics falls away? A positive answer to this question is fundamental to what can be called a *strong conception* of introspection. In contrast, a *weak conception* of introspection only takes introspective results as preliminary results that are to be investigated further based on larger sample sizes yielding statistical significance. On the one hand, the strong conception of introspection seems far-fetched – intersubjective agreement can hardly be abandoned outright – on the other hand, the weak conception of introspection only gives us an explorative method. We can ask, however, whether there is a domain between these two extremes. Can we develop introspective methods that are more than explorative and provide results that can be attributed with a moderate, if not mathematical, level of certainty?

If we look to the history of philosophy, we can indeed find claims stating that introspective insight is the only reliable type of insight; it is the only kind of insight that is truly indubitable. Descartes’ statement *cogito ergo sum* is a famous example, though the truth of this claim has been contested ever since it was uttered. A recent defense of the certainty of *cogito ergo sum* was formulated by Jaako Hintikka, who interpreted the statement as pointing to the insight that one cannot *perform the act* of doubting that one exists without at the same time *confirming to oneself* that one exists (Hintikka, 1962). However, it has been more common to take a critical stance in relation to introspection. As already mentioned, introspection encountered fundamental problems early on in its development, and has continued to be challenged. A typical example of this is a study done by Nisbett and Wilson (Nisbett and Wilson, 1977), which showed that we often make mistakes when we investigate our own decision making process, and the criticism of introspection continues today (Schwitzgebel, 2008; Smithies, 2013). Some research exists, showing that introspection may become more reliable when it is done methodologically through for instance micro-phenomenology (Petitmengin et al., 2013; Sparby et al., 2021), Although micro-phenomenology is mostly conducted by guiding others to investigate their experience systematically, it may also be done in a way where a person investigates their own experience (Sparby et al., 2020b; Sparby, 2023) and methodical advancements like relying on a cultivation of the sense of certainty may improve the reliability of reports (Sparby et al., 2020a). Still, the empirical evidence that it is possible to improve the reliability of reports is scarce.

Hence, one may describe the current situation like this: introspection is, by some, counted as the most secure source of knowledge, others view it as very unreliable. Tim Bayne has already identified this state of affairs, referring to the former as optimists and the latter as pessimists with regards to the status of introspection (Bayne, 2015). Some forms of introspection seem more reliable than others. For example, to use Bayne’s terminology, *scaffolded introspective judgments* seem more reliable than *freestanding* ones. My introspective judgment that I currently am having a visual experience of a red tomato is *scaffolded* by there being a red tomato in front of me. Internal phenomena, such as a decision process, do not rely directly on sensory support and introspective judgments about such processes are known to be unreliable (Nisbett and Wilson, 1977; Petitmengin, 2006).

However, even if introspection is sometimes unreliable or even if there are cases that are difficult to decide introspectively, this does not mean that introspective methods are in principle problematic. Indeed, sensory perception is often known to be unreliable – even systematically unreliable given the right conditions – which has been shown very clearly through different examples of perceptual illusions, for example in relation to lightness perception (Adelson, 2000). And yet it would not occur to us to claim that perception should be dismissed in general.

To illustrate in more detail: An interesting case is provided by Wittgenstein, who speaks of a kind of arithmetic of color, i.e., a form of knowledge of the essence of colors, a phenomenology that can be developed by simply looking at and reflecting on the nature of colors. For instance, Wittgenstein states that white will be the brightest color in any picture (Wittgenstein, 2007, p. 17). It seems hard to disagree. Similarly, a pure blue will always be darker than a pure yellow (Wittgenstein, 2007, p. 17). If we have compared colors like that once, we know that for any similar pair of blue and yellow, blue will be the darker color. Hence we can, with the help of single phenomena, uncover necessary truths. In relation to other colors, however, it is not so clear. Which of the colors red and blue are darker? Violet and blue? Even though there are some cases that are unclear, this does not mean that all are, and it certainly does not mean that sensory information is always unreliable. In the same vein, though introspection may in some cases be unreliable, this does not mean that it always will be. There may be cases where we can uncover necessary truths by investigating phenomena introspectively. Again, this does not mean that we can or should abandon intersubjectivity; in particular in cases where getting clear introspective results is challenging, seeking intersubjective confirmation is valuable in that it provides a general safeguard against human fallibility. Easy cases of introspection, like whether I feel warm or cold in a daily life context does not need external confirmation, but for investigating complex topics such as freedom and attention within a scientific context, critical intersubjective exchange does provide a way of challenging, and thereby securing, the accuracy and universality of reports and hence takes a definite methodical step toward making the results more certain. Other ways of increasing reliability include using micro-phenomenology, This will be addressed again in the discussion.

### 1.2 Free will and attention

“Free will” is a very broad topic and has been debated for centuries. A distinction can be made between freedom in the sense of being *able to decide* and *being able to act* (Keil, 2017), which can also be called a distinction between internal and external freedom. A person can deliberate and make a decision to move from A to B while at the same time not being able to do so because some external obstacle, like a closed door, stands in the way. In such a case, the person would be inwardly free but still not free to carry out their will externally. In the debate about free will, it is usually internal freedom that is cast into doubt.

Though most will agree that it indeed *seems* that we are free, it is a currently widespread view that free will is an illusion. For instance, recent studies suggest that the sense of having made a choice, i.e., having committed a freely willed action, may be a *post hoc* construction (Wegner and Wheatly, 1999; Bear and Bloom, 2016). The “experience” of freedom can then be understood as being produced by subconscious processes in the mind or the brain (Wegner, 2003), a position that remains controversial (Bayne, 2005; Carruthers, 2007). Hence, describing the *phenomenology* of freedom, i.e., the way freedom appears, might very well be compatible with both affirming and denying that we are actually free. However, denying free will despite the appearance of the opposite will require an additional explanation as to how and why the illusion of freedom appears. Affirming that free will exists faces the problem of explaining how a physical universe can accommodate non-physical powers or abilities. These are all complex topics that cannot be settled here. Though it common to deny that free will exists, there is work currently being done in philosophy that supports the view that free will is real. J.T. Ismael recently suggested that physical laws can be considered to be similar to the laws that regulate how chess pieces are moved around on a chess board while playing chess (Ismael, 2016); the laws limit the ways the pieces can move, but do not indicate how they will actually move within those boundaries. Furthermore, Steward explores the Aristotelian option of anchoring freedom in the capacity for self-movement (Steward, 2012), and such concepts as self-movement can be helpful in analysing freedom, regardless of whether or not agency really exists. There are also deep and complex issues involved in defining and realizing freedom comprehensively. Freedom does not necessarily involved being *unlimited*, but can involve the limiting of, for example, the realization of one’s desires and rather “binding” oneself to the dictates of reason (one’s own nature) (Kant, 2012). Indeed, one can even speak of a dialectic of freedom in relation to limitations and going beyond them, which is vital to consider when speaking of the social and spiritual realization of freedom (Sparby, 2016). Here we are primarily interested in the experience of freedom and hence we can bracket both the issue of whether (internal) freedom is real and also the more complex issues relating to the realization of freedom. However, a theory of freedom that does not want to end up with an epistemological dualism – in which appearances never accord with how things really are – must show how the apparent experience of freedom can be reconciled with the facts of reality. For instance, if it is impossible to have direct access to the source of action through a widening of attention, this reconciliation will remain incomplete; the experience of committing an act would be forever separate from the one who commits it and hence we could never have certain knowledge of our freedom. This will be discussed in section 4.1.

Attention can both be directed and sustained. We can direct attention toward an object or a series of objects, and we can choose to hold our attention steady on an object, which is the same as sustaining attention. Sustaining attention on the same object for more than a short moment is generally more difficult than directing attention. Sustaining attention is bound to lead to mind-wandering. Consequently, it seems that we have more control over directing attention than sustaining it.

Attention itself can be said to be free to the extent to which it can be linked to the capacity of self-movement. Directing attention seems to be a clear manifestation of the ability of self-movement and therefore also of freedom. We can move our body, which is a manifestation of the power of self-movement, but if we stop moving the body, it stays in place. This is different from how self-movement manifests in attention. Attention does not stay in one place, if left to itself. Hence we have to extend the notion of freedom to include the ability for a person to resist movement, if they so choose to. It is not hard to sustain attention for a short time, but the challenge grows as the time span of the task becomes longer. When mind-wandering occurs, this is a form of unintended movement of attention. Thus, sustaining attention can only be said to be a realization of freedom given the condition that an intention to sustain attention over time is present. Without being able to sustain and control attention, without having meta-awareness or being mindful of what we are doing in the moment and whether what we are doing is in accordance with our ideals, we are severely limited in the way we can express our freedom. As Metzinger has pointed out, our freedom or autonomy is limited by our lack of control over our attention and thoughts (Metzinger, 2013). It may seem that Metzinger intends to argue that cognitive agency is a complete myth, that all mental action is determined by subpersonal processes. However, his claim is that we do not have mental autonomy, including control of our attention, for roughly two thirds of our lives. Metzinger’s idea of mental autonomy, or M-autonomy, is based on the notion that we are only able to act according to our ideals and hence to express our nature as rational creatures to the extent that we can maintain awareness of our ideals and cognitive agency over time. Increasing control over attention and mind-wandering can therefore be seen as a precondition of the realization of freedom, and the practice of attention tasks such as meditation and mindfulness can be viewed as different means of increasing freedom, as these contribute to the control and stabilization of attention and decrease of mind-wandering (Feruglio et al., 2021). Metzinger also accepts the idea that meditation is a way of systematically cultivating M-autonomy.

Does directing attention always involve sustaining attention, even if for a short time, at specific places for instance in the sensory field? Is it possible to sustain attention without continually directing it toward an object? These are examples of the kind of empirical questions that introspection can help shed light on. However, the research literature on attention is vast, and there are numerous issues in which introspection does not stand immediately at the foreground. Wu has suggested that there are five central questions in the field of attention (Wu, 2014):

1. The metaphysical question: What is attention?
2. The question of function: What role does attention play?
3. The question of properties: What are characteristic features of attention?
4. The question of mechanism: How is attention implemented?
5. The question of consciousness: What is the relationship between attention and consciousness?

With a little reflection, however, we can see that introspection would always, at least tacitly, be involved in answering these questions. Investigating the mechanism of attention, for instance, looking at how attention is related to human physiology, the eyes, the brain, and so on, would depend on correlating experiential reports with physiological data. The quality of the correlation not only depends on the accuracy of the physical measurements, but also on the accuracy of the reports. Since there are no established means assuring the quality of introspective reports, current research on attention could be viewed as inherently flawed. The current study is an initial investigation into how introspective methods can increase the accuracy of first person reports and hence quality of attention research.

Furthermore, Dicey Jennings has pointed out (Dicey Jennings, 2014) that although Wu mentions subject-centred or phenomenological approaches, his list lacks the question of the *source* of attention. What or who directs and sustains attention? Can we speak of an agent in relation to this? What is the experience of this source? Is it possible to direct attention toward the source or can it only be explored indirectly? This is another example of how introspection can become more explicitly involved in the research on attention. For instance, before one can even begin to look for neurological correlates of the source of attention, the source of attention will first have to be described, and a consensus about the meaning of this term in all its facets will have to be reached. Some subject-centred approaches to attention and agency do exist (Depraz and Depraz, 2004; Gallagher, 2012; Jennings, 2012), but they have not addressed the question of the source of attention through methodical introspection, as attempted in the present study.

To summarize, the primary questions of this study are: What is the phenomenology of directing and sustaining attention? How does directing and sustaining attention relate to freedom? What is the phenomenology – if any – of experiencing the source of attention?

## 2 Method

This study involved six participants. Their academic background ranges from philosophy, psychology and physics to medicine and aesthetics. All have an expertise in different forms of attention practices, such as meditation, precise observation of patients, and active perception of art. Most participants offer different forms of training in these fields and are also colleagues working together on different research projects. During an initial meeting, the topic was discussed, and different forms of introspective tasks and practices for directing and sustaining attention were explored. It was agreed upon to direct and sustain attention using a real apple seed, then to use an imagined representation of that apple seed, and finally to see whether it is possible to direct attention toward the source of attention and sustain it there. An apple seed was chosen since it is a visually simple object that is relatively easy to represent in imagination and also easily accessible. After 2 weeks the participants met again to discuss their experiences. It became clear that certain tasks had been performed differently by the participants and that some clarification was necessary to establish a consensus about how to actually perform the practices. In particular, three clarifications were made: *Sustaining* attention consists of focussing one’s attention on an object without thinking about the object; the task should be conducted with both a real and an imagined object; an attempt should be made to direct and sustain attention at the source of attention after working both with a real an imagined object. This resulted in the following eight tasks:

1 With a real apple seed
a Direct attention toward the seed
b Sustain attention on the seed
c Direct attention toward that which directs and sustains attention
d Sustain attention on that which directs and sustains attention

b With an imagined apple seed
1 Direct attention toward the seed
2 Sustain attention on the seed
3 Direct attention toward that which directs and sustains attention
4 Sustain attention on that which directs and sustains attention

These tasks were practiced for another 3 weeks. The reported duration of practice ranged from 5 min working on a single task and up to a total of 1 h and 15 min for completing all tasks. During these 3 weeks the participants also shared and reflected on their experiences among themselves. All participants met again for a final time to present and discuss their results. Some time was spent on discussing theoretical aspects relating to freedom and attention, and some on comparing experiences, uncovering which of them were the same or similar and which were different, opposite, or unique. Due to the complexity of the reports and issues involved, and to allow close analysis and comparison, it was agreed that the participants were to submit written reports to one of the participants, who would then conduct a thematic analysis (Guest et al., 2012) assisted by an analysis-software (MAXQDA), which was conducted by the first author. In a final meeting with three participants, the analysis was presented and the results were discussed. After this meeting the reports were read through once more, a few further experiences were identified, the identification of specific text passages was revised, and the categorical structure was finalized. In some cases where the reports were unclear, the participants were asked to elaborate. All participants had the chance to check the presentation of the results before the analysis was completed.

## 3 Results

The analysis resulted in 669 text passages being identified and this formed the basis for creating a taxonomy of the reported experiences. Common experiences (experiences reported by four or more participants) will be presented in 3.1 as well contrary reports in 3.2. When reports are quoted, the participant number is stated within the brackets following immediately after the quoted text. A full taxonomy of the experiences is provided in the Supplementary file S1.

### 3.1 Common experiences

Common experiences are experiences that are reported by four or more of the participants and relate to the following topics: Agency, distractions, mind-wandering, effort, insight, affects visual experiences, arousal, and difficulties.

#### 3.1.1 Agency

All participants reported on different aspects of agency, which relates to the sense of self described above. In particular, four participants stated that one’s activity or intentionality is experienced indirectly through conducting the tasks. As one participant states: “It is clear to me that I generate activity all the time [2].” Another participant elaborates after having conducted task 1c (directing attention toward that which directs and sustains attention):

First I asked myself: Where I am? Or who am I within this activity, within this act of perception? I am not the apple seed and all its properties, but rather that which determines the direction, modality, and movement. “I” experience “myself” as the one who acts through changing the aspects, which is initiated “through” “me”. I experience my self through the “expected” change that is “induced”. It is an experience of a self-activity happening through intentionally changing what is perceived [5].

The emphasis here is that the self is experienced through perceptual change and the intentional change that lies behind that.

Two participants reported that agency is only noticed retrospectively, i.e., after having conducted a task and reflecting on who is conducting the activity. Again, further results pertaining to this will be presented below. One participant notes that discovering one’s agency can also be connected to a sense of joy, though, as another participant states, conducting the tasks can also, because mind-wandering keeps occurring, be experienced as discovering the limits of one’s agency. As one participant notes: “The question of who directs attention in relation to the representational task [i.e. 2c] makes the answer “I” appear. But that is initially just a word; what is behind it is to begin with not possible to experience [3].” This statement opens up the discussion about whether it is possible to experience the source, which will be treated in section 3.3.1 and 4.1.

#### 3.1.2 Distractions

There were four participants reporting on distractions, either specific ones or in general. Distractions can be either external (e.g., noises in the environment) or internal (e.g., inner images), and they can be of comparatively stronger and weaker degrees. In some cases, the participants were aware that something, such as the feeling that the body is cold, is a distraction; in others cases, the awareness that something is a distraction was not present, like when an association has presented itself in consciousness (which can lead to mind-wandering, see below). Distractions include: Memories, associations, things one has to take care of in daily life, inner issues with which one is concerned, bodily sensations (muscle tension, pain), noises in the environment, internal talk, insights, and internal quasi-visual shapes.

One participant reports that a lack of activity or focus can lead to becoming distracted: “Additionally, it can happen, when there is too little activity and focus, that I “slip off” into the nearest environment and find new “interesting” things there [2].” Another participant notes that distractions can come either as separate or combined elements. For instance, a series of memories that are visually represented inwardly can appear in combination with internal talk, forming a narrative. When the distractions only contain one element, they were stated to be easier to notice.

#### 3.1.3 Mind-wandering

All participants reported on episodes mind-wandering. Mind-wandering can be distinguished from distractions in that mind-wandering consists of a series of connected mental events arising from a distraction. Here is an example of a report of a mind-wandering episode: “There was a moment of mind-wandering as the memory appeared of how my father handled the food and the apple seeds [1].” And another: “For example, when inwardly constructing the brown object that is the apple seed, it happens that I, accompanied by the thought “brown as a hazelnut,” think “hazelnut, hazelnut cream, hazelnut cream during the visit to the family of my brother last weekend”; and then I was suddenly within the world of memories associated with this weekend [5].” The types of mind-wandering events mentioned are fantasies, streams of associations, memories, and reflections (one participant notes that it is possible to mind-wander even when the eyes are open and the gaze fixed). Indicators of mind-wandering come from the different dimensions of experience already mentioned:

1. Level of Consciousness: Tiredness (low degree of arousal)
2. Cognitive: Looking for the source of attention, transitions within an overarching task, surprises, thinking without visual content, forgetfulness
3. Imagination: Quick images that pull attention away
4. Perceptual: Staring
5. Affects: Curiosity, fear, lack of interest, irritation, a wish to do something else (boredom)
6. Volition: Lack of effort, or that the intention for doing the tasks is not internal to the subject
7. Somatic: Bodily sensations

Certain more or less effective anti-dotes to mind-wandering were also noted:

1. Performing the attention task with a physical rather than inwardly represented object
2. Consciously calming down
3. Giving oneself a command, or pulling oneself together, or activating the will
4. Remaining in continual attentional activity
5. Not giving in to impulses

Five participants note that remaining in continual attentional activity works well as an antidote. However, there is no guarantee that mind-wandering is reduced over time even when conducting concentration tasks with strong effort.

#### 3.1.4 Effort

Four participants report on different aspects of effort. The attentional tasks mostly require continual effort; the inner image, for instance, rarely stabilizes and becomes continuous. One participant, however, reports the following: “The image of the apple seed […] has to be recreated in every moment and this requires significant effort; rarely there is an experience of a continuity of the image beyond a few moments. But when it happens, it is a gift and is experienced as a liberation [3].” In other words, even though the experience of stabilization is rare, when it happens, it can be a very positive experience.

#### 3.1.5 Insight

Four participants describe different ways in which they gain insight into the experience of freedom or autonomy or start questioning whether or in what way we really are free. One participant describes an insight into how freedom is enacted through being able to both direct attention and re-direct after becoming distracted. Another participant, however, identifies a split in consciousness: “There is some resistance to going back to the object. How is this autonomy then? Immediate wishes and set intentions are separate; I identify with one former on a surface level and the latter on a deeper level [6].” Thus the participant questions whether directing attention is necessarily a case of freedom or autonomy in that there is a conflict between the intention to focus and the wish to not do so.

#### 3.1.6 Affects: Joy

The one emotion that is mentioned most often (by five participants) is the emotion of joy (other emotions are mentioned three times or less). The experience of joy is connected to either the sense of being active, to the stabilization of attention, or sustaining attention at the source.

#### 3.1.7 Visual experiences

Reports on visual experiences relate to either aspects of imagination and perception or to a domain between both where imaginal and perceptual elements can intermingle.

A common experience is that it can be challenging to construct and sustain an inner image. As one participant notes, the sensory concreteness of the inner image is missing. Another participant notes that the inner image might resist being shaped as intended or even that the image starts to shift its shape spontaneously. The only common perceptual report is that distortions that happen when attention is sustained on a real apple seed. The impression can become blurry, a whiteness can appear around the seed, or the visual field itself becomes blank or grey. In contrast, the reports about the domain that lies between imagination and perception is quite rich; a light can be experienced, flickering or shimmering phenomena can appear, there can be a play of after images, or other impressions of colors and shapes appear in a quasi-visual field.

#### 3.1.8 Arousal: tiredness

Five participants describe experiences relating to tiredness. Three note that tiredness makes the concentration tasks difficult, for instance through mind-wandering becoming more prominent, and one participant reports having fallen asleep during the task.

### 3.2 Contrary reports

There were contrary reports in relation to the central question of whether it is possible to experience the source of attention. The reports can be divided into claims that it is possible or impossible to direct and/or sustain attention at the source and that it is easy or difficult. The claims that it is easy or difficult are subcategories of the claims that it is possible. Furthermore, there were some contrary reports relating to positive and negative affect, and to the level of consciousness.

#### 3.2.1 It is possible to direct and/or sustain attention at the source

Some participants report being able to direct attention to the source by directing attention to the activity that is involved in conducting a task. A participant states: “One can become aware of the source through the “mirror” of the objects; the activity of the observer mirrors itself in the constant change in the aspects of the perceived object [5].” Participant [2] has a similar observation:

To direct attention towards that which directs and sustains attention. That is for me now and then a completely delightful state: to notice that it is me – my own activity that brings forth the image of the apple seed inwardly. At least it is clear to me that only that happens, which is really intended by me – otherwise not much would happen and the image of the apple seed would not appear. How the processes take place on a more detailed level and who directs them, how they are directed and how calm re-enters, to that I can say nothing yet. That escapes the capacity of observation which I currently possess [2].

Hence it is stated that the source can be observed, though what is observed is the activity of bringing forth the image. Note, however, that the participant implies that it could become possible to say more about some parts of the process of attending to an object, and about the source, through an improvement of the capacity of observation.

One participant states that it is impossible to direct attention to the source while observing an external object:

The question about who directs perception towards the apple seed was not possible to treat while the perceptual task was being done, it could only be done subsequently. Then, however, the immediacy of the perceptual activity was no longer present; one had, to put it like this, extrapolate it from memory, and, doing this, it became apparent that one had to use an image of the apple seed as support. Then I had, however, proceeded from the perceptual task to the imaginal task. It was not clear to me how I could have done the task differently [3].

Thus it seems possible to direct attention toward the source, but only in retrospect. Another participant reports that it is possible to direct and sustain the attention on the source while conducting the task with an external object, but notes thcat it seems more difficult:

It seems even more difficult to find the source of the activity of directing and sustaining attention when the eyes are open. The body seems to stand more in the focus. When looking for the source, I direct attention to the eyes, towards the body. Maybe this is because there isn’t much activity involved in fixing the gaze? You just hold the eyes at one place; it isn’t really hard. When shifting the gaze I certainly go to the eyes if I try to locate the source of the activity. When moving the eyes around quickly it is easier to notice that it is me who’s doing it. Then it also becomes clear again that attention is different from vision; I can move my eyes around while my attention is elsewhere. A frustration arises from not finding the source – not getting a sense of how I am directing my gaze – while it still seems so obvious that it is I am moving the eyes/the gaze [6].

The same participant reports that directing and sustaining attention on the source is not only possible, but also easier than working with an inner image: “I can direct attention to the source of that which creates the image without re-creating the image. [This is] [e] asier than creating an inner image [6]”; “[It is] [m] uch easier to focus on the source. The sense of the source seems immediate. [6]”; “It seems very easy to rest in the source of the activity of directing attention, but it is not possible to locate the existence of the source anywhere in space [6].” Here is a similar also slightly more elaborate account:

Directing and sustaining at the source: There is an inner vibrancy, clarity. But also an emptiness. Inner shimmering or flicker. Light (vague). Noticing a separation between the sense of activity and the source; the activity is more connected to the body/the sense of vibration. The source feels deeper, more “inward” and backward at the same time, like it’s in another kind of space, but I’m losing orientation when looking for it. A sense of relief of not having to do a task. This is much easier than imaging an image [6].

To summarize, there are a range of claims relating to the possibility of either directing attention to the source or sustaining it there: Such attention (i) is possible; (ii) is possible and even easier than creating an inner image; (iii) is difficult, for instance while the eyes open; (iv) is only possible in retrospect; (v) is possible through noticing who is performing the attentional activity.

#### 3.2.2 It is impossible to direct and/or sustain attention at the source

It has already been indicated that some participants connect the possibility of being aware of the source of attention with being active. A further participant states: “I was not able to rest within the source while completely forgetting the apple seed [4].” Another participant offers the following remark:

To rest within the continually active going-out-of-oneself (the source of the activity) is not possible. […] to “rest” within that which directs attention, i.e. that which is the origin of attention, is not possible. One would have to duplicate oneself. One can try to direct the attentional direction to […] the represented object and at the same time to the creation of the representation. But also then the activity shows itself in the feeling of what has been done by oneself and in the change of the formation of the image [5].

And elaborates:

To rest in that which directs attention (towards the apple seed): Observations: I cannot direct perception towards the source of the continual change and movement of the attentional direction; I can only try: in doing that the apple seed (the observed object) fades somewhat. When doing that, I enter into non-objectivity. The source is continual activity, which tries to grasp itself.

[…] To direct attention towards that which directs and makes attention rest: As already stated, the productivity and its source can be experienced more strongly in the production of an image than in external (sense-)perception. Likewise, the source cannot be viewed as an object [5].

Here the point is not only that the source can only be brought into view through being active, but also that it is impossible to see the source as an object.

#### 3.2.3 Positive and negative affect

It can also be noted that although five reports contain references to joyful feelings that can be experienced while conducting the task (and three note a calming affect), there are a few cases of negative affects, such as resistance, aversion, or strain. One report states: “I experience resting [attention] on this image as completely artificial, extremely strenuous, und not really attainable [1].” The same participant connects this to the fact that the intention was not really internal, i.e., it originated in another person or a group of persons (i.e., the research group). One participant notes the contrast between positive and negative affect explicitly: “There’s a theme of frustration with losing the object of attention, on the one hand, and joy and happiness that comes as a result of working with attention like this, on the other [6].”

#### 3.2.4 Level of consciousness

One participant notes that the task itself can result in tiredness. In contrast, one participant reports: “Sometimes the mind stabilizes; everything becomes more clear and I feel more awake.” It might not be surprising that conducting a task can be tiring, but that it can lead to becoming more awake, can be viewed as significance. Possible explanations for this will be discussed below.

## 4 Discussion

The results will first be discussed in relation to the question of whether it is possible to introspect in the source of attention (4.1). Then issues relating to freedom and the stabilization of attention in general will be discussed (4.2), followed by some remarks on the methodical aspect of introspective research and contemplative science (4.3).

### 4.1 The source of attention

The most central issue in relation to freedom that has arisen from this study is whether it is possible to experience the source of attention directly or only indirectly, i.e., through or while being active. This issue has been discussed in philosophy at least since Kant (1904) and Fichte (Fichte, 1971), and it has been recently treated by Strawson (2010), 2015). In Kant’s view, since the subject could only appear to itself as an object, it is impossible for it to appear to itself as it is in itself. This echoes one of the reports of this study: “Likewise, the source cannot be viewed as an object [5].” Arguing against this view, Fichte claimed that the self can be present to itself immediately in an act of intellectual intuition:

This intuiting of himself that is required of the philosopher, in performing the act whereby the self arises for him, I refer to as *intellectual intuition*. It is the immediate consciousness that I act, and what I enact: it is that whereby I know something because I do it. We cannot prove from concepts that this power of intellectual intuition exists, nor evolve from them what it may be. Everyone must discover it immediately in himself, or he will never make its acquaintance. The demand to have it proved for one by reasoning is vastly more extraordinary than would be the demand of a person born blind to have it explained to him what colors are, without his needing to see (Fichte, 1982, p. 38)

Self-consciousness is, by Fichte, conceived as an activity that can perceive itself, or, more precisely, the activity of self-consciousness and the perception thereof “together form a single essence” (Prager, 2010, p. 9). The idea of a pure activity goes back to Aristotle, who argued that “within the series of things which are intelligible *per se*, absolute primacy is given to the kind of substance which is completely simple and in a state of pure activity (Clearly, 1995, p. 398). For Aristotle, pure activity is exhibited by the highest being, i.e., God or the unmoved mover (Aristotle, 1984, pp. 1071b5–1073a1), but it can also be achieved by contemplation, which is the “purest of all activities” and “the most final good” (Korsgaard et al., 1996, p. 239). And as indicated, Strawson has recently explored the claim that the subject can “take itself as it is in the present moment of awareness as the object of its awareness” (Strawson, 2011, p. 274); Strawson offers a theoretical argument for the possibility of such a form of awareness, but notes that the actual experience of it requires a “sort of meditative condition”:

[…] it’s simply a matter of coming to awareness of oneself as a mental presence (or perhaps simply as: mental presence) in a certain sort of alert but essentially unpointed, global way. The case is not like the eye that can’t see itself, or the fingertip that can’t touch itself. These old images are weak. A mind is rather more than an eye or a finger. […] It’s a matter of first focusing on the given fact of consciousness and then letting go in a certain way. As far as the level of difficulty is concerned, it’s like maintaining one’s balance on a parallel bar or a wire in a let-go manner that is relatively but not extremely hard to attain. One can easily lose one’s balance—one can fall out of the state in question—but one can also keep it, and improve with practice. (Strawson, 2011, pp. 292–293)

Strawon claims that not only is it theoretically possible for the subject to be present to itself as it is in the present moment, but also that this is a task that is achievable; though it is perhaps difficult initially, the ability to remain in such a state can be trained and in the end it might even become easy.

The pure present moment self-awareness of a subject can possibly be interpreted as pure activity. Though such an interpretation will have to be worked out in more detail, the idea of pure activity can potentially resolve the issue of the conflicting reports. Discovering and attending to the source of attention more fully may be an issue of having developed a certain capacity, which is also implied in one report (“That escapes the capacity of observation that I currently possess”). Several participants noted that being active is a requirement for noticing who or what is being active. This does not mean, however, that the source of attention is *not* itself an activity; it could be a form of pure, and *simple* activity. If it is a pure and simple activity, this can explain why it is at once difficult to reach while at the same time it is relatively easy to attend to as soon as it is discovered. A pure activity is different from any other activity, in that it does not relate to an activity external to the subject itself – hence it may require a difficult and unfamiliar form of attention. When pure activity is discovered, however, it becomes easy to sustain attention on it, since this does not require one to make use of any external sense organs or mental capacities external to the subject; it remains, simply, present to itself. To make use of an image of Aristotle (1984, p. 1071b11): The self-awareness of the source may be represented as a circular motion, like a stream flowing continuously and continually around in a circle, where the singular moments of awareness turns into an uninterrupted flow of an overarching awareness. Whether or not this is in accordance with actual experience, i.e., whether attending to the source is an experience of pure activity and whether such a form of attention is trainable and can potentially become easily available, cannot be decided based on the basis of the present material.

A problem of conflicting introspective reports that mirrors the one discussed above has been encountered within the field called cognitive phenomenology. Cognitive phenomenology asks whether cognition has a certain quality or qualia, a “what-it-is-likeness,” just like tasting an ice cream is experienced in a certain way. The problem encountered in cognitive phenomenology is that some people claim that cognition is connected to unique class of qualia, while other do not (Bayne and Montague, 2012). The reason for this may be that only some people have the capacity of experiencing this kind of qualia. Furthermore, theoretical presuppositions may also lead to either connecting qualities of experiences to thinking that really are disconnected or a failure to identify such connections. Similarly, directing and sustaining attention at the source may be a capacity that can be developed, claims to the effect that it is possible to attend to the source may either wrongly connect certain subtle qualities to the source or rely on a failure to identify an underlying activity that mediates the experience, or it might be the case that theoretical presuppositions involving the notion that it is impossible to attend to the source may block the actual experience of it.

Again, these issues cut to the core of the methodological foundation of introspection and presents challenges to be met by future studies and theoretical work. The question of whether it is possible to attend to the source immediately remains open. Even if it is true that the source of attention can only be attended to indirectly, i.e., through an activity that is reflected upon after the activity has come to an end, this does not make future introspective research on the source of attention futile; it would, however, need to take into account that what the source is cannot be experienced directly. And if the source cannot be experienced directly, this opens up for the possibility that conscious experiences, such as memories of previous events, are indeed constructions, produced either by subconscious processes or the brain, and hence subjective. Although such a reductive position can never be confirmed directly either, simply because it involves the claim that everything in consciousness is always mediated by something external to it, the result is in either case an epistemological impasse that cannot be overcome unless a way is found to justify that it is possible to have an immediate access to consciousness as it is in itself.

### 4.2 Freedom and the stabilization of attention

As described earlier, being able to direct and sustain attention is arguably a condition of freedom (Metzinger, 2013). The research literature also distinguishes “captured attention” from consciously directed attention (Wu, 2014); insofar attention is captured, freedom is also limited. We can also conceive of a state in which attention is captured sustainedly. Examples of this are trance-states that cannot be consciously exited. Are such trance states the result of successful meditative stabilization of attention? If so, full stabilization of attention would actually be contrary to freedom; freedom requires the ability of shifting attention to any object of choice. Even though in a trance state a wish to do something else might not be present, the inability of exiting the trance, i.e., directing attention elsewhere, means that freedom is inhibited. There are, however, deep meditative states in which stabilization is complete, while the ability of exiting the state is still present (Snyder and Rasmussen, 2009). Hence such a full stabilization of attention would be compatible with free will, but only when the condition of being able to exit the state at will is met.

### 4.3 Remarks on the introspective research method and contemplative science

The intention of this study was to bring actual introspective reports to bear on the issue of free will and the possibility of experiencing the source of attention. Other methods of first-person, introspective research such as micro-phenomenology (Petitmengin, 2006) exist and have been used to explore the process of stabilization of attention (Sparby, 2019a,b). While the present article adds to the content of the introspective research, one of its main contributions is methodical: How does one approach the topic when a group of researchers are involved who take equal part in the introspective research process? A main challenge is the existence of conflicting reports on fundamental issues. This may make it less likely to become convinced of the potential of involving introspective reports in the discussion of epistemological and ontological problems. There are different ways of approaching this. One way (1) is to make theoretical prejudice clear at the outset of a study and make an effort to bracket such prejudice while introspecting. This can counteract cases where prejudice actually influences experience or the descriptions of it in such a way to make it contradict the reports of others with a different theoretical outlook. A second (2) way is to consider whether certain experiences depended on a certain level of expertise. If so, it may be necessary to develop more precise roadmaps of how to access such experience in order for others to be able to investigate them. A third (3) way is to consider whether conflicting reports may be describing different aspects of the same phenomena and whether some concepts can be found that unify the descriptions.

This study has revealed an example of an approach that combines 2 and 3: We suggest that directing attention toward its source and sustaining it there may be a case of maintaining attention within an essence that consists of simple and pure activity. This form of activity may be difficult without practice. It may be noted that this is a theoretical suggestion that will have to be investigated further, but it also gives a direction for future studies. Consequently, a productive way of dealing with disagreement is to device further empirical tasks, informed by one or more of the approaches (1–3) that were just mentioned.

In general, the way to move from a weak conception of introspection – i.e. one that only results in a preliminary investigation – to a stronger conception, is to make specific methodical suggestions for how to address concrete issues or problems that are already known or arise in the course of a new study. In general it can be recommended to conduct studies over a long time period during which tasks are repeatedly performed, as this can reveal both invariant structures and variable characteristics of phenomena. Moreover, it is advisable to conduct introspective studies in groups that engage both empirically and reflectively with the phenomena, since this not only helps counteract fallibility and one-sided theoretical viewpoints, but also begins to reveal which structures may be invariable across larger populations.

Introspective results become more strongly convincing when the conceptual structures or conditions of a phenomenon are clearly and simply formulated. An example of this would be Husserl’s idea of protention and retention as acts of consciousness that are a necessary condition for the experience of music (Husserl, 1986). To experience, for instance, an interval, requires that the first tone is both anticipated to continue while the second tone appears and is retained while the second notes resounds. While this may be convincing on a theoretical level, one may still ask: Is this actually what musical experience is like? Hence, conceptual and empirical investigations can mutually support each other. Carving out the nature of something at its joints through an introspective study, revealing for instance the basic structure and features of an emotion, will provide convincing results not only because it manages to unite a series of subordinate features through the identification of underlying structures, but also because people actually experience the emotion in question like this and can potentially learn more about their experience by considering it in light of the introspective results.

A final way of making introspective results more secure is triangulation. Triangulation in this context means to relate certain introspective results to other domains of knowledge, which is also a basic dimension of what scientific activity consists of (Hoyningen-Huene, 2016). One example is to correlate a statement indicating a specific level of arousal with relevant biological measurements. For instance, decreased breathing might be an indicator of low arousal. However, triangulation can always be challenged based on either source of empirical data. Slowed down breathing or even breath cessation may in some cases indicate high arousal, as evidence by some studies on deep meditative states (Badawi et al., 1984; Travis and Wallace, 1997). In such cases, an introspective report of high arousal may be challenged by the exhibited behavior, but the interpretation of the behavior may with equal justification be challenged by the introspective account. This shows the limit of triangulation, but problems like this may also result in developing a better understanding both of behavior and first-person experiences, as it may lead to investigating the phenomena more closely and refining our understanding of behavior: Is the arousal that arises in conjunction with breath slowing down somehow experienced differently from the arousal that arises on conjunction with increased breath rate? Is the former correlated with different physiological markers of arousal than the latter? These questions, and the way one would have to go about answering them precisely, shows the relevance of introspective research within a larger context of scientific conduct.

We have given an example of how contemplative first-person research may proceed in a group setting in a way that sheds light on contemporary issues in psychology and philosophy. It has become clear that introspective observation, although essential, is only one part of the process. Equally important is the conceptual work done in relation to the results of the introspective observation. This kind of work becomes especially relevant when the introspective observation shows different results: Is this due to individual differences? How does one argue that one result is false and the other correct? Are there ways of conceptual integration that make it possible to explain different observational results?

Traditionally, contemplative practice has been focused more on general structures of meditative development irrespective of individual differences and less on the details of what goes on in the mind of individuals, including disagreements of experiential reports. Contemplative science that involves first-person reports now has a chance of remedying this by including introspective methods in group settings. While this kind of work is time intensive and cognitively challenging, it is not different from other fields of science in this regard. What is different in the form of contemplative science that we outline here, is the requirement of the involvement of the whole person; one is not only studying one’s own experience in-depth, but also one’s own experience in relation to the experiences of others. This expands the purely introspective approach to include an intersubjective aspect (Weger and Wagemann, 2015b; Trnka and Smelik, 2020), which is fundamental to the scientific pursuit of truth.

## 5 Conclusion

This study shows the potential for using introspection in conjunction with conceptual analysis to study central issues within psychology and philosophy, such as attention and freedom. An overview of the different aspects of the phenomenology of attention has been developed, including common experiences, contrary cases, and significant single cases. Though freedom can be experienced through the activity of directing and sustaining attention, a consensus about whether or not it is possible to experience the source of attention did not arise. However, the different descriptions resulted in a tension that provoked deeper theoretical reflection, and it was suggested that the notion that the source consists of pure activity might unify the different viewpoints that were expressed. This gives a direction for future introspective research on the topics of freedom and attention.

## Data availability statement

The datasets presented in this article are not readily available because the data belongs to the respective authors. They may provide their own data individually upon request. Requests to access the datasets should be directed to terje.sparby@gmail.com.

## Author contributions

TS: Conceptualization, Investigation, Methodology, Writing – original draft, Writing – review & editing. DC: Conceptualization, Investigation, Methodology, Writing – review & editing. DH: Conceptualization, Investigation, Methodology, Writing – review & editing. FE: Conceptualization, Investigation, Methodology, Writing – review & editing. DT: Conceptualization, Investigation, Methodology, Writing – review & editing. UW: Conceptualization, Investigation, Methodology, Writing – review & editing.
