# Supplementary material for: The phenomenology of attentional control: a first-person approach to contemplative science and the issue of free will
Source: Front Psychol. 2024 Mar 12;15:1349826. doi: 10.3389/fpsyg.2024.1349826 (PMC10963616; doi:10.3389/fpsyg.2024.1349826)

## Taxonomy

This taxonomy gives an overview of the actual reports, i.e. it shows the *range of experience* rather than how often they occurred. The main categories of the taxonomy are: 1. Level of Consciousness. 2. Sense of self. 3. Cognition. 4. Perception-imagination axis. 5. Sensation. 6. Affect. 7. Volition. 8. Somatic. 9. Dynamic or static forces. 10. Difficulties. An overview of the taxonomy with one of three sublevels is presented by figure 1 below.

### 1 Level Consciousness

Level of consciousness refers to experiences relating to different degrees of wakefulness and includes daydreaming.

### 2 Sense of Self

The sense of self, which also includes the sense of agency, could either increase or decrease as part of conducting the tasks. The decrease was in one case connected to lending “some of my sense of self to the image so that it gains more subsistence, more of a continuity, increasing in qualities such as clarity and stability [6]”, i.e. in other words the decrease lead to the task being conducted more successfully. An increase in the sense of self can happen in the form of a resistance to having the object of attention take up the whole stream of consciousness. Further experiences relating to the sense of self, in particular with regard to the experience of agency when focusing attention, will be presented under common experiences (3.2.1). “Sense of self” also contains reports relating to the source of attention, such as statements about whether it is possible or impossible to experience the source (see 4.1).

### 3 Cognition

The category of cognition contains five sub-categories: Attention, memories, thoughts, meta-awareness, and insights, which will be described in more detail:

#### 3.1 Attention

Attention consists of three further sub-categories: Form, distractions, and mind-wandering. The category of form contains all statements relating to directing, re-directing, and sustaining attention, but also other observations such as that attention is like an instrument that one can make use of. Distractions contain the types of distractions and whether they are weak or strong. The category of mind-wandering contains statements of the intensity and duration of mind-wandering while the participants were conducting the tasks, as well as the types, indicators, anti-dotes and examples of cessation of mind-wandering.

#### 3.2 Thoughts

This category contains descriptions of different forms of thought-related experiences that occur during the attention tasks, such as thoughts being automatically generated.

#### 3.3 Memories

Some of the reports about memories are directly related to the task, for instance using memories to construct the image of the seed, but also include participants noting that old memories appear while conducting the task and that it can be difficult to remember everything that transpired during the task.

#### 3.4 Meta-awareness

This category contains reports about the mental faculty that notices that one is no longer conducting a task.

#### 3.5 Insights

Insights relate to knowledge that appears as the task is performed. These can either be insights into task-related aspects (such as the nature of attention) or completely unrelated.

### 4 Imagination-Perception Axis

This category contains the subcategories of “auditory” and “visual”. Some of these experiences are imaginations, others are perceptions. However, certain experiences lie somewhere in the middle of an axis consisting of perception (which is more passive/receptive or objective) as one extreme and imagination (which is more active or subjective) on the other. Some of the experiences that lie in the middle, such as after images, are in a sense objective without being perceptions, since they are not created by a conscious act of inner representation but also do not relate information about a world external to the subject. Other experiences in the middle of the axis are more subjective, such as when a quasi-visual field or quasi-visual representations are experienced; these are neither imaginations nor clear sensory impressions. Similarly, auditory experiences include “white noise” and a quasi-auditory sound of suction. Again, these experiences can be designated “quasi” because they are not identified as perceptions while they still have a more objective character than an inner representation.

### 5 Sensation

There were many different sensations reported as well: General intensification of sensations, vibrancy, presence, flickering, clarity, emptiness. Sometimes these are referred to simply as “subtle qualities” and sometimes they are metaphors (“emptiness”) or qualities that could belong to specific sense modalities. Clarity could belong to either visual or auditory perception, but when none of these are specified, they have been categorized as “sensation”.

### 6 Affect

Experiences of a number of emotions were reported: Expectation, enthusiasm, relief, joy, devotion, gratitude, peace, confidence, frustration, aversion, resistance, fear, curiosity, and boredom.

### 7 Volition

This category contains statements about free will and effort. Some participants report an increase in the awareness of freedom (which is an insight), but also notice how the experience of freedom itself can either increase or decrease during the tasks. One participant reports freedom increasing by not giving in to an impulse that would mean suspending the task, while another reports that the task itself appears pointless and therefore is experienced as a decrease of freedom. Effort contains observations on when attention requires more effort, the necessity of continually exerting effort, or episodes of effortlessness.

### 8 Somatic

Many somatic experiences were reported, including: Tingling, disembodiment (reduced sense of having a body or out of body experience), contracting muscles, respiration increasing or decreasing, different tactile experiences, energies in the body, and gestures.

### 9 Dynamic and Static Forces

This category contains reports describing forms of contracting and expanding forces, such as vibration, pressure, and radiation.

### 10 Difficulties

Reported difficulties include finding enough time to do the tasks, laziness, actually understanding what the task consists of, difficulties conducting the tasks because of external distractions, failing to remember what actually happened while doing the tasks, boredom, and frustration. This conflict between positive and negative affect, which was reported on more occasions, will be discussed in section 4.3.

Figure 1: Overview of Taxonomy


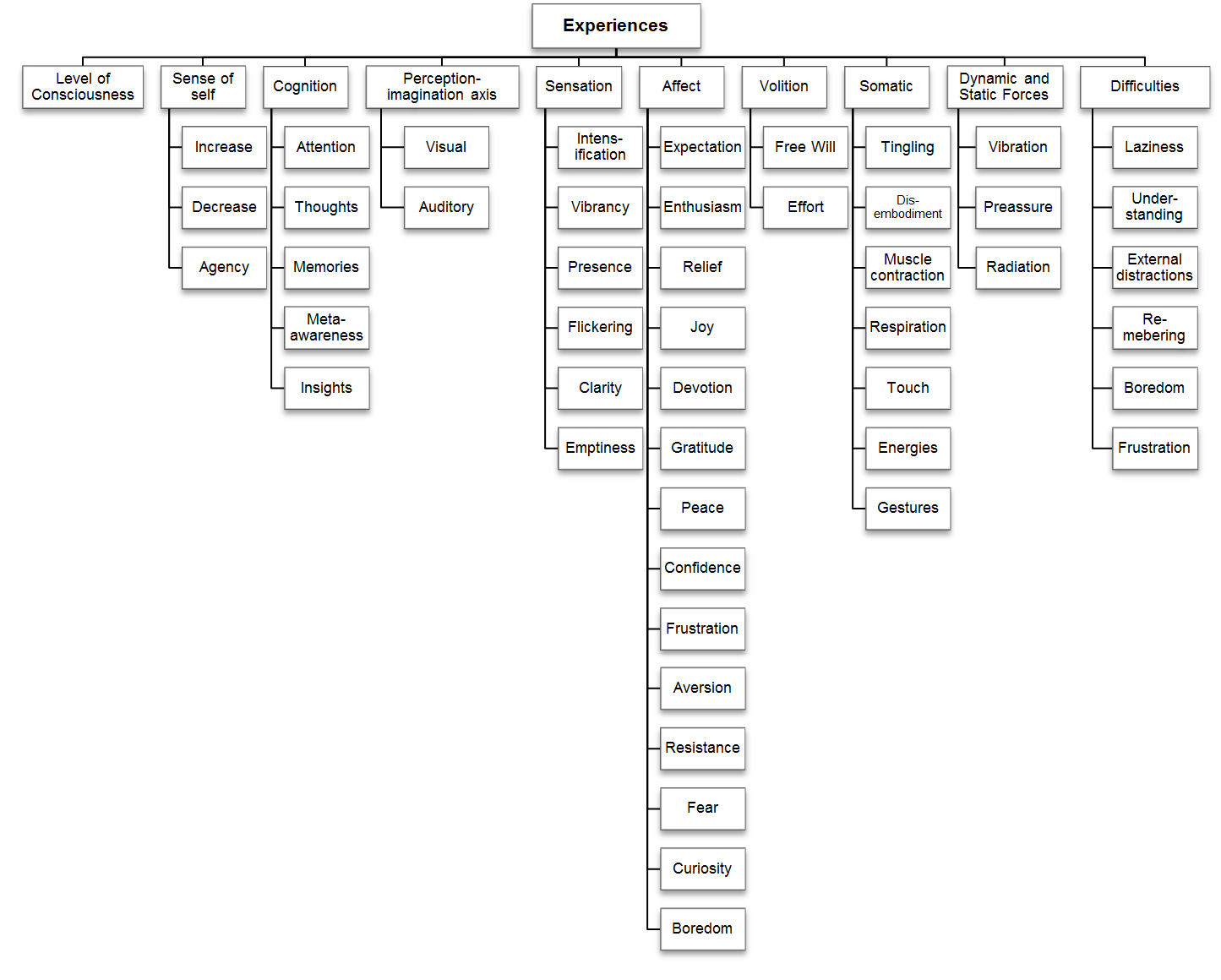

Supplement: Supplementary file 1 [file Table_1.DOCX]
